# The Latest Look at PDT and Immune Checkpoints

**DOI:** 10.3390/cimb46070430

**Published:** 2024-07-08

**Authors:** David Aebisher, Agnieszka Przygórzewska, Dorota Bartusik-Aebisher

**Affiliations:** 1 Department of Photomedicine and Physical Chemistry, Medical College, The Rzeszów University, 35-959 Rzeszów, Poland; 2 English Division Science Club, Medical College of The Rzeszów University, 35-025 Rzeszów, Poland; ap117623@stud.ur.edu.pl; 3 Department of Biochemistry and General Chemistry, Medical College of The Rzeszów University, 35-025 Rzeszów, Poland; dbartusikaebisher@ur.edu.pl

**Keywords:** PDT, immune checkpoints, effectiveness, cancer cell

## Abstract

Photodynamic therapy (PDT) can not only directly eliminate cancer cells, but can also stimulate antitumor immune responses. It also affects the expression of immune checkpoints. The purpose of this review is to collect, analyze, and summarize recent news about PDT and immune checkpoints, along with their inhibitors, and to identify future research directions that may enhance the effectiveness of this approach. A search for research articles published between January 2023 and March 2024 was conducted in PubMed/MEDLINE. Eligibility criteria were as follows: (1) papers describing PDT and immune checkpoints, (2) only original research papers, (3) only papers describing new reports in the field of PDT and immune checkpoints, and (4) both in vitro and in vivo papers. Exclusion criteria included (1) papers written in a language other than Polish or English, (2) review papers, and (3) papers published before January 2023. 24 papers describing new data on PDT and immune checkpoints have been published since January 2023. These included information on the effects of PDT on immune checkpoints, and attempts to associate PDT with ICI and with other molecules to modulate immune checkpoints, improve the immunosuppressive environment of the tumor, and resolve PDT-related problems. They also focused on the development of new nanoparticles that can improve the delivery of photosensitizers and drugs selectively to the tumor. The effect of PDT on the level of immune checkpoints and the associated activity of the immune system has not been fully elucidated further, and reports in this area are divergent, indicating the complexity of the interaction between PDT and the immune system. PDT-based strategies have been shown to have a beneficial effect on the delivery of ICI to the tumor. The utility of PDT in enhancing the induction of the antitumor response by participating in the triggering of immunogenic cell death, the exposure of tumor antigens, and the release of various alarm signals that together promote the activation of dendritic cells and other components of the immune system has also been demonstrated, with the result that PDT can enhance the antitumor immune response induced by ICI therapy. PDT also enables multifaceted regulation of the tumor’s immunosuppressive environment, as a result of which ICI therapy has the potential to achieve better antitumor efficacy. The current review has presented evidence of PDT’s ability to modulate the level of immune checkpoints and the effectiveness of the association of PDT with ICIs and other molecules in inducing an effective immune response against cancer cells. However, these studies are at an early stage and many more observations need to be made to confirm their efficacy. The new research directions indicated may contribute to the development of further strategies.

## 1. Introduction

**Figure 1:**
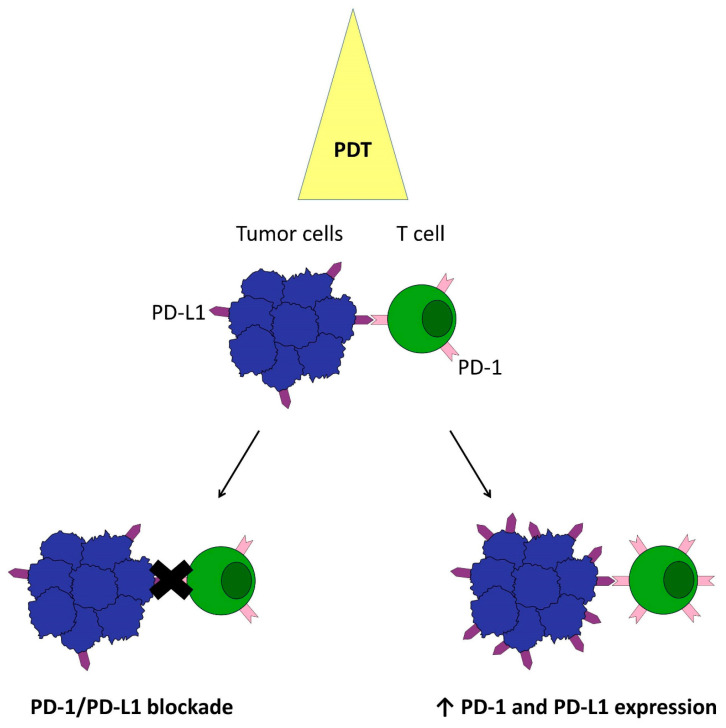
Effects of photodynamic therapy on the PD1/PD-L1 axis. (1) PDT with PS chlorin e6 through an unexplained mechanism has the ability to block the PD-1/PD-L1 axis and increase CD8+ T-cell activity, but without inducing an antitumor immune response [31,32,33,34,35]; (2) ALA-PDT can increase PD-1 expression on lymphocytes, while Redaporfin-PDT can increase PD-L1 expression on tumor cells [36,37]. The impact of these changes on the effectiveness of therapy is described further in the text.

Cancer is one of the common diseases with a high mortality rate worldwide and is the most important obstacle to improving the overall life expectancy of the population in the 21st century [1]. New therapeutic options are constantly being sought to improve this situation. In this regard, photodynamic therapy (PDT) has attracted increasing attention in recent years due to its potential in cancer treatment [2,3,4]. Although PDT results vary depending on the type of cancer, minimal tissue toxicity, low systemic effects, high efficacy, and minimal side effects make it a valuable therapeutic option [5]. PDT has been clinically approved for the treatment of head and neck cancer, esophageal cancer, pancreatic cancer, prostate cancer, and squamous cell carcinoma [6]. PDT uses three main agents: a photosensitizer (PS), light, and oxygen [7,8]. Absorption of light by the photosensitizer molecule causes it to go from a ground state to a short-lived excited singlet state. An intersystem transition then occurs to the more stable excited triplet state. From this triplet state, the photosensitizer molecules can return to the ground state through a type I or type II photodynamic reaction. In a type I reaction, the activated photosensitizer transfers an electron to the substrate to form various reactive oxygen species. In a type II reaction, energy is directly transferred to molecular oxygen in the ground state, resulting in the formation of highly reactive singlet oxygen [9]. It is widely believed that singlet oxygen is the main agent responsible for the photodynamic destruction of cells and tissues. Most of the PSs currently in use work primarily through a type II mechanism [10]. Reactive oxygen species kill tumor cells, destroy tumor blood vessels, and lead to tumor regression through cell death mechanisms [11]. Depending on the subcellular localization of the photosensitizer, cell membrane damage, or disruption of mitochondria, endoplasmic reticulum or lysosomes can occur. This in turn triggers stress reactions, leading to cell necrosis, apoptosis, or autophagy [12]. Photosensitizer photoactivation promotes the detachment of endothelial tissues from the basement membrane of blood vessels. This creates thrombogenic areas characterized by the aggregation of thrombocytes, production of vasoactive molecules, and increased permeability and vasoconstriction [13]. This leads to cancer cell death due to a lack of oxygen and nutrition, thus inhibiting cancer growth [14]. PDT also stimulates the immune response. Destruction of cancer cell organelles and membranes activates phospholipases and cyclooxygenases, leading to the release of inflammatory mediators that attract leukocytes that fight cancer cells [15]. However, the antitumor effects induced by PDT are influenced by several factors, including PS localization in cells, PS concentration, light fluence rate, oxygen concentration, and integrity of immune function [16]. Therefore, PDT still has many limitations, such as metastatic tumors, insufficient light delivery, and a lack of adequate oxygen [17]. As a result, PDT is limited to treating thin and superficial tumors. Recently, however, emerging nanotechnology has contributed to advances in many areas, including PDT, improving its ability to penetrate deep tumor tissues and produce the desired therapeutic effects [10,18]. In addition, photodynamic therapy can not only eliminate cancer cells, but can also stimulate antitumor immune responses [19]. Results have shown that PDT at a high dose of light, as a therapeutic dose, induced early immune activation followed by late immunosuppression, which was mediated by activated TGF-β1 regulation [20]. Also, photogenerated cytotoxic molecules localized in tumor tissue can lead to immunogenic cell death (ICD), which releases damage-related molecular patterns and tumor-specific antigens [21]. The field of cancer photodynamic therapy, like oncology research in general, is showing increasing interest in cancer immunology and the immunological effects of cancer treatment [22]. Immunotherapy has made tremendous clinical progress in recent years [23]. However, it is only effective in a fraction of cancer patients due to low response rates and serious side effects. Findings indicate that the challenges of immunotherapy in the clinic can be solved by inducing immunogenic cell death, among others. ICD is currently considered one of the most promising ways to achieve complete eradication of cancer cells [24]. It is induced to release threat-associated molecular patterns (DAMPs) and tumor-associated antigens to facilitate dendritic cell (DC) maturation and infiltration of cytotoxic T lymphocytes (CTLs). This process can reverse the immunosuppressive tumor microenvironment (TME) and significantly improve therapeutic efficacy [25,26]. Consequently, immune checkpoint blockade is now one of the most important types of antitumor immunotherapy, which aims to restore the function of effector immune cells, most commonly T cells, by administering antibodies that block inhibitory molecules [27]. It has been shown that the efficacy of PDT and immunotherapy in preventing cancer metastasis and recurrence can be significantly improved primarily through combined strategies [28,29]. Synergistic enhancement of ICD may be particularly important in this area [30] (Figure 1).

## 2. Methods

**Figure 2:**
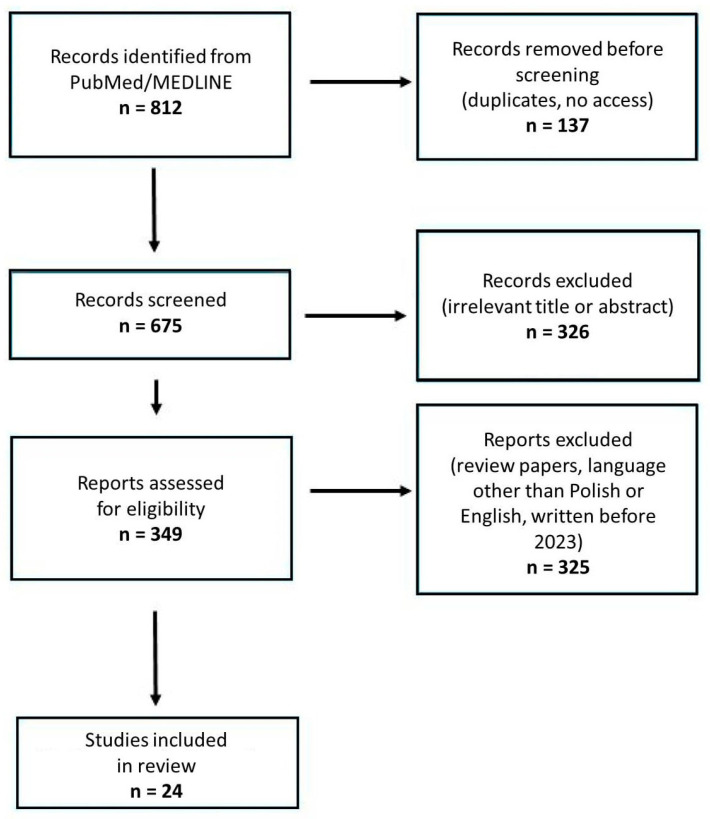
PRISMA flow diagram of the studies included.

A literature search that focused on papers describing photodynamic therapy and immune checkpoints in the treatment of malignant tumors was conducted using the PubMed/MEDLINE database. The following terms were searched: “PDT AND immunotherapy”, “PDT and immune checkpoint inhibitors”, and “PDT and immune checkpoint” (Figure 2). A total of 812 papers were identifed. Only research papers, both in vivo and in vitro, were eligible. To describe recent developments, articles written before January 2023 were excluded. Only articles written in English or Polish were eligible. The final number of papers selected was 24. The inclusion and exclusion criteria are shown in Table 1.

## 3. Results and Discussion

### 3.1. PDT versus Immunological Checkpoints

Programmed cell death protein 1 (PD-1) and programmed death-ligand (PD-L1) are typical immune checkpoints that transmit inhibitory signals to silence host immunity [31,32]. The up-regulation of PD-L1 in tumor cells has been shown to be generally associated with tumor progression, proliferation, and invasion; anti-apoptotic signaling; and T-cell inhibitory activity through PD-1 engagement [33,34]. Reducing their negative effects is particularly desirable in cancer therapy. A number of recent studies describe the potential of PDT to improve the efficacy of the immune response by affecting immune checkpoints. Work by Gurung et al. demonstrates that PDT with PS chlorin e6 through an unexplained mechanism has the ability to block the PD-1/PD-L1 axis and increase CD8+ T-cell activity. However, they observed that despite the increased infiltration of these cells in some of the mice tested, they were depleted and an antitumor immune response was not induced [35]. On the other hand, Lobo et al. and Anand et al. show that in some tumor cells, PDT increased CTLA-4 and PD-L1 and PD-1 expression on lymphocytes, as schematically shown in Figure 1 [36,37]. However, this may have a potentially beneficial effect on immune checkpoint inhibitors (ICI) therapy, as pre-existing T cells in the tumor are a prerequisite for response to anti-PD-1 therapy, and the lack of reactive PD-L1 expression may indicate a poor response to PD-1 blockade therapy due to impaired T cells infiltrating the tumor [38]. Moreover, it has been found that PDT can stimulate cells in some tumors to exhibit high levels of CD80 molecules on their surfaces. Overexpression of CD80 enhances tumor immunogenicity [36]. The formation of a cis-PD-L1-CD80 duplex, by binding CD80 to PD-L1, attenuates PD-L1-PD-1 binding and abrogates PD-1 function [39]. Removing this PD-1 restriction has been shown to have an immunosuppressive effect and may be effective in treating autoimmune disease [40]. The cis-PD-L1-CD80 duplex also inhibits CTLA-4 inhibitory receptors binding to CD80 in cancer cells [36]. Delcanale et al. point out that there does not appear to be a correlation between PDT efficacy and PD-L1 expression levels, indicating that mechanisms other than simple receptor density determine treatment outcome [40]. In conclusion, reports on PDT’s ability to modulate PD-1 and PD-L1 levels lead to conflicting conclusions, and the mechanism is not completely elucidated.

### 3.2. Association of PDT with Immune Checkpoint Blockers

Checkpoint-blocking immunotherapy has recently been successful in the clinic, but elicits only limited rates of systemic antitumor response for most cancers due to insufficient activation of the host immune system [41,42]. Many reasons for this phenomenon have been identified [34,43,44,45]. Photodynamic therapy-based combination therapies have gained much attention in various cancers due to their potent therapeutic effects [46]. These include induction of immunogenic cell death, production of damage-related molecular patterns and tumor-associated antigens, and reprogramming of the tumor microenvironment [47]. All of these processes promote the activation of antigen-presenting cells and promote T-cell infiltration, so combining ICI treatment with PDT can address the reasons for the ineffectiveness of ICI therapy alone and enhance the antitumor immune response leading to effective immunotherapy, as we describe below [42,48,49].

#### 3.2.1. Abnormal Tumor Vascularization

**Figure 3:**
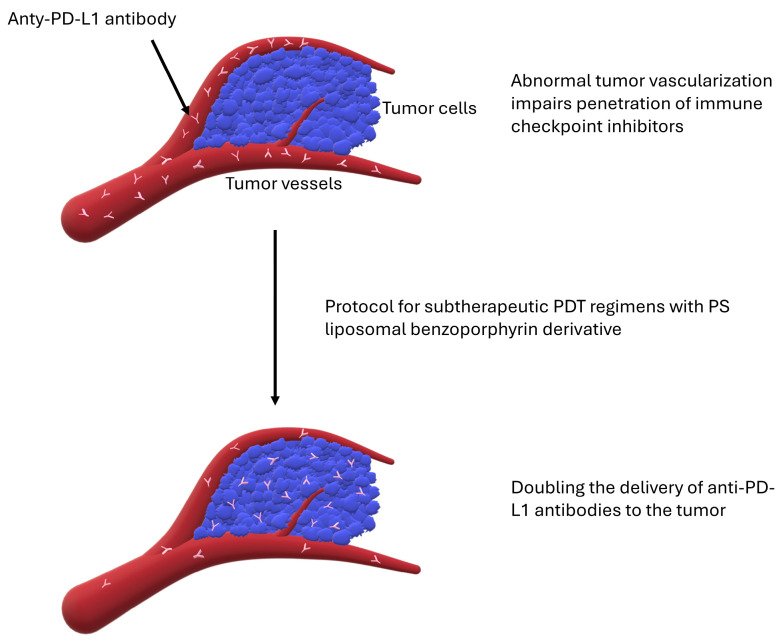
Abnormal tumor vascularization impairs the penetration of immune checkpoint inhibitors. It can be doubled by a regimen of subtherapeutic PDT with PS liposomal benzoporphyrin derivative [52].

Increased angiogenesis in TME, the resulting abnormal vascularization, and high interstitial pressure within the tumor can impair the penetration of checkpoint inhibitors [50]. Moreover, endothelial cells may express PD-L1, which may further impair T-cell function in the tumor microenvironment [50,51]. In recent years, PDT has been found to have the ability to affect the biochemical and physical properties of TME [52]. It has been shown that by damaging endothelial components, PDT can significantly increase vascular permeability (Figure 3). However, these same effects can also lead to platelet aggregation, coagulation, and reduced blood flow in the tumor area [53]. In their novel work, Bhandari et al. doubled the delivery of ⍺-PD-L1 antibodies in AT-84 mouse head and neck tumors using a single protocol of subtherapeutic photodynamic therapy regimens utilizing a liposomal benzoporphyrin derivative. Moreover, this improves the immune response by inducing immunogenic cell death and a 3–11 fold increase in tumor cell exposure to damage-related molecular patterns [52]. Zheng et al. developed a new nanoparticle (Combo-NP) consisting of a biodegradable pseudo-conjugate of the fluorescent polymer NIR II with disulfide bonds in its main chain and the vascular inhibitor lenvatinib [54]. Lenvatinib is a multi-kinase inhibitor that targets VEGFR1/2/3, FGFR, PDGFRB, KIT, and RET [55]. In a number of studies, lenvatinib in combination with PD-1/PD-L1 signaling inhibitors showed more potent antitumor activity than either drug alone [56]. Clinically, lenvatinib in combination with pembrolizumab has also shown promise, such as in the treatment of endometrial cancer [56,57]. Photodynamic therapy with Combo-NP led to remodeling and normalization of the tumor vasculature, thus enhancing the efficacy of PDT while promoting increased lymphocyte infiltration in the tumor microenvironment. Moreover, it also induced immunogenic cell death. In addition, in combination with anti-PD-L1, it showed a significant inhibitory effect on tumor metastasis [54].

#### 3.2.2. Immunosuppressive Cells

The immunosuppressive effect of the tumor microenvironment is also characterized by the presence of cells with a suppressor function against functional immune cells, such as Tregs, myeloid-derived suppressor cells, and tumor-associated macrophages (TAMs) [38,45]. These may be responsible for the failure of immune checkpoint blocker therapy [58,59]. Recent studies have shown that human TAMs also express PD-1, which increases with increasing disease stage, and exhibit an M2-like surface profile. PD-1 expression on PD-1+TAM is associated with decreased macrophage phagocytosis and increased tumor cell invasion, which can lead to poor cancer prognosis [43]. Moreover, it has been found that tumor-associated macrophages can be a major source of PD-L1 [60]. Recruitment of M2 macrophages thus appears to lead to resistance to immunotherapy by affecting tumor angiogenesis, invasion, metastasis, and immunosuppression [34,61]. In the context of reducing the negative impact of M2 macrophages on ICI efficacy, the work of Sun et al. is interesting. They designed extracellular vesicles (EV) expressing Siglec-10 previously obtained from 4T1 breast cancer cells, additionally combined with a photosensitizer-loaded lipid nanosystem [62]. Siglec-10, a member of the sialic acid-binding immunoglobulin superfamily, has been identified as an immune checkpoint that is expressed on macrophages in various types of cancer [63,64]. The interaction of this molecule with CD24 on the surface of cancer cells can activate SHP-1 and/or SHP-2 phosphatases associated with tyrosine inhibitory motifs (ITIMs), which prevents TLR-mediated inflammation and cell engulfment by macrophages [65,66]. By combining Siglec-10 from nanoparticles with CD24 of cancer cells, blocking Siglec-10 attachment of macrophages was achieved increasing their anti-inflammatory activity. The generation of ROS by the photosensitizer during PDT can convert immunosuppressive M2 macrophages into M1 macrophages [62]. The EVs contained all tumor antigens, thus directly activating dendritic cells and a specific immune response [62]. In summary, this approach not only activated the antitumor immune response, but also reduced the negative impact of the immunosuppressive tumor environment on the effectiveness of the immune response. A new strategy for effective immunotherapy against “cold” tumors was developed by Wang et al. The molecule they created, with activity similar to a number of enzymes, including superoxide dismutase, catalase, peroxidase, and glutathione oxidase, solved a number of problems associated with tumor immunosuppression. It promoted M2 to M1 macrophage polarization, alleviated hypoxia in TME, generated ROS, and depleted glutathione in TME to expose necrotic cell fragments and reverse immunosuppressive TME by inducing dendritic cell maturation and infiltration of cytotoxic T cells in tumors [67].

Indoleamine 2,3-dioxygenase (IDO) 1, IDO2, and tryptophan 2,3-dioxygenase (TDO) are responsible for degrading tryptophan to its downstream metabolites. Decreased tryptophan levels and increased IDO1 expression correlate with increased levels of immunosuppressive cells such as regulatory T cells and myeloid-derived suppressor cells, decreased levels of tumor infiltrating lymphocytes and NK cells, and up-regulation of PD-1 in cytotoxic T cells [68]. Thus, it seemed natural to combine IDO inhibitor therapy with immune checkpoint blockers. To date, however, the results of clinical trials using indoleamine 2,3-dioxygenase inhibitors and anti-PD-1 have been disappointing [68,69]. Importantly, IDO1 expression levels vary between tumor types and among patients with the same tumor type, which may account for treatment failures [68]. An attractive strategy to improve this association was developed by Zheng et al. They created a self-validating biomedicine based on the photosensitizer chlorin e6, an IDO inhibitor (NLG919), and a PD-1/PD-L1 blocker (BMS-1). PDT with this molecule led to significant cell apoptosis and ICD, while NLG919-induced IDO inhibition and BMS-1-induced PD-1/PD-L1 blockade improved the immunosuppressive tumor microenvironment to enhance the immune activation cascade. The ultimate effect was inhibition of tumor growth and metastasis [70]. In conclusion, improving the immunosuppressive tumor microenvironment and reducing the negative effects of immunosuppressive cells can enhance the efficacy of treatment with immune checkpoint blockers in several ways.

#### 3.2.3. Loss of Tumor Neoantigens and Insufficient Stimulation of the Antitumor Response

It has been shown that loss of neoantigens, which are antigens encoded by the mutant gene of cancer cells, may be the cause of the ineffectiveness of anti-PD-1 therapy [38]. Emerging research supports the critical role of neoantigens in the response to PD-1 blockade therapy [71]. Evidence available to date suggests that chemotherapy promotes tumor antigen release and antigen presentation and stimulates immune factors [38]. Combining checkpoint inhibitors with chemotherapy has proven clinically effective in the treatment of triple-negative breast cancer [72]. The addition of ICD-stimulating PDT to these treatments may therefore be an attractive form of enhancing the immune response. Zhang et al. designed spherical nucleic acids based on the PD-L1 aptamer, consisting of a porphyrin Zr6 MOF core and PD-L1 aptamer shell, with oxaliplatin encapsulated in MOF nanopores. The combination of PDT, chemotherapy, and immunotherapy provided effective induction of immunogenic cell death and activation of the antitumor immune response. Moreover, precise delivery of the immune checkpoint inhibitor enhanced the immune response and minimized systemic toxicity [73]. Also, Feng et al. associated PDT with chemotherapy and immunotherapy. They created mitochondria-targeted nanoparticles capable of improving the efficacy of PDT and delivering the active metabolite of irinotecan SN-38. These particles induced immunogenic cell death and promoted the recruitment and activation of cytotoxic T cells. They were also shown to enhance the antitumor efficacy of anti-PD-1 antibody in a mouse model of a CT26 tumor [74]. It can be speculated that PDT played a key role in the effective immune response, as it was previously shown that the response to irinotecan-based chemotherapy after PD-1 blockade in patients with advanced esophageal squamous cell carcinoma appeared similar to that previously observed in patients who did not receive PD-1 antibodies [75]. A paper by Yao et al. designed a prodrug consisting of acid-activated doxorubicin and an aggregation-induced emission photosensitizer combined with a caspase-3-responsive peptide, and showed that the combination of repetitive PDT with anti-PD-1 therapy further promoted proliferation and intratumor infiltration of cytotoxic T cells and effectively inhibited tumor growth and lung metastasis [76]. Clinical data available to date indicate that short-term doxorubicin therapy can induce a more favorable tumor microenvironment and increase the likelihood of response to PD-1 blockade in triple-negative breast cancer. However, these data require confirmation [77]. It has been observed that treatment of breast cancer with doxorubicin induced up-regulation of IDO1, so it seems interesting to attach an IDO1 inhibitor to this strategy [78].

**Figure 4:**
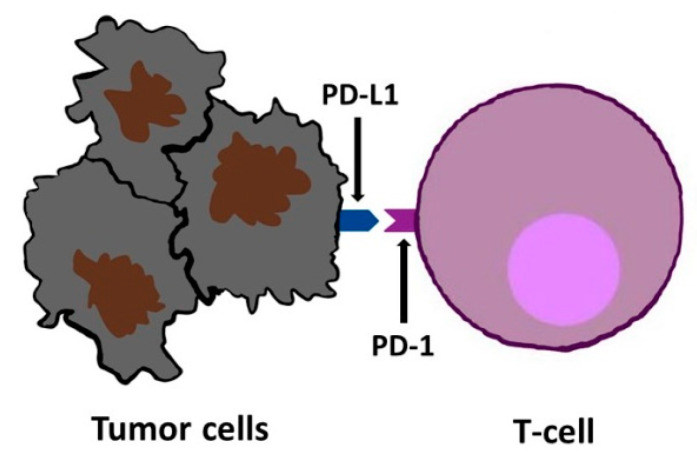
Interaction between PD-1 localized on a T lymphocyte and PD-L1 of a cancer cell.

Induction of pyroptosis and activation of the cyclic guanosine monophosphate/adenosine synthase/interferon gene stimulator (cGAS/STING) signaling pathway may be a new strategy to break the immunosuppressive tumor environment and improve the efficacy of anti-PD-L1 immunotherapy [79]. Recent studies have demonstrated the feasibility and clinical potential of using pyroptosis as a mechanism of antitumor immunity. Pyroptosis can increase the immune response and enhance the effect of immunotherapy through the release of various tumor antigens, DAMPs, and pro-inflammatory cytokines [80,81,82,83]. A limitation of this approach, however, is that pyroptosis can be strongly correlated with the proliferation and migration of various tumor cells [80,81]. The cyclic GMP-AMP synthase (cGAS) and stimulator of interferon genes (STING) pathway has emerged as a critical innate immune pathway [84]. As a DNA receptor, cyclic GMP-AMP synthase recognizes abnormal DNA in the cytoplasm and activates STING. This signaling cascade leads to an immune response produced by type I interferon and other immune mediators, but in some cases chronic STING activation can promote tumorigenesis [85]. STING agonists have been shown to reduce the side effects of drugs and overcome tolerance to cancer treatment [85]. It is also known that immune checkpoint blockade therapy remains limited by an insufficient immune response in “cold” tumors [67]. To address this problem, Yang et al. developed albumin nanospheres with photosensitizer-IR780 encapsulated in the core and cGAS-STING agonists/h2S-ZnS producer placed on the shell. Their results suggest that treatment based on this molecule can remodel the TME by increasing infiltration of CD8 + T cells and decreasing immunosuppressive Treg cells, thus changing triple-negative breast cancer from a “cold” tumor to a “hot” tumor, and thereby improving the efficacy of anti-PD-L1 immunotherapy [79]. Moreover, intracellular zinc ions can produce ROS, which can compensate for the diminished effect of PDT by tumor hypoxia [79,86]. Qu et al. developed a nanoparticle targeting TME of pancreatic ductal adenocarcinoma (PDAC) through tumor-specific midkine nanobodies, which can effectively deliver solid-state polymer nanoparticles to TME PDACs and locally produce abundant reactive oxygen species for precise photoimmunotherapy [87]. PDACs are among the prototypical immunogenically “cold” tumors because they lack the natural infiltration of ICI-responsive effector T cells. It has been found that in order to transform PDACs into ICI-responsive tumors, effective immunotherapy strategies are required to increase antigenicity, enhance T-cell function, and overcome T-cell exclusion and immunosuppressive signals in the tumor microenvironment [88]. PD-1 was shown to be highly expressed in the PDAC TME and was present on 64% and 71% of CD4+ and CD8+ T cells, respectively [89]. Available data indicate that both of these problems can be addressed by PDT. A novel approach by Qu et al. induced immunogenic cell death by remodeling immunosuppressive TME with increased T-cell infiltration. Combined with PD-1 checkpoint blockade, the targeted PDT platform significantly prolonged mouse survival [87]. Tumor immunogenicity was augmented by Su et al. by creating nanoparticles that respond to the tumor microenvironment and near-infrared light to degrade endogenous tumor H2O2 and the ability to deplete glutathione (Figure 4). They show that the use of this molecule with anti-PD-1 can be used to eliminate primary tumors and inhibit the growth of untreated distant tumors and cancer metastases [90].

It has been shown that induction of ferroptosis can overcome resistance to immunotherapy with checkpoint blockers, as it can produce a vaccine-like effect to stimulate antitumor immunity [91,92]. The association of PDT with ICI is therefore particularly attractive because of PDT’s ability to induce this cell death [93]. Induction of feroptosis, however, may be prevented by ferroptosis suppressor protein 1 (FSP1) [94]. Zhou et al. developed novel molecules containing FSP1 and the photosensitizer chlorin e6 showing high efficiency in triggering ferroptosis and immunogenic cell death, both in vitro and in vivo. They also observed increased tumor infiltration by CD8+ T cells and efficacy of anti-PD-L1 immunotherapy [93]. On the other hand, increased ROS production in TME can lead to localized neutrophil ferroptosis and release of oxidized lipids that limit T-cell activity [95,96]. In the work of Zhu et al., for the first time, PDT was combined with the ferroptosis inhibitor liproxstatin-1 and ICI. The enhanced photodynamic therapy, mediated by di-iodinated IR780 (Icy7), significantly increased the production of reactive oxygen species with induction of immunogenic cell death. At the same time, liproxstatin-1 effectively inhibited ferroptosis of cancer neutrophils. Ultimately, this strategy enhanced anti-PD-1 treatment [97].

A novel approach to producing tumor-associated antigens and improving PD-L1 efficacy features in the work of Gu et al. They constructed a molecule that contains the photosensitizer IR780, 2,2′-azobis [2-(2-imidazolin-2-yl)propane]-dihydrochloride (AIPH), and an anti-PD-L1 antibody [98]. The use of AIPH enables oxygen-independent generation of nitrogen gas and ROS under thermal excitation, so it can overcome hypoxic TME [99]. This possibility is particularly interesting because hypoxia can induce PD-L1 expression [100,101]. This nanosystem has been shown to effectively inhibit both primary and distant tumors [98].

Through a novel approach, Zhao et al. achieved an effective immune response and elimination of cancer metastasis. They designed a nanoparticle that contains the photosensitizer chlorin e6, an ASCT2 inhibitor (V9302), and a PD-1/PD-L1 blocker [102]. V-9302 is a transmembrane glutamine flow antagonist that selectively and potently targets the amino acid transporter ASCT2. The pharmacological blockade of ASCT2 with V-9302 resulted in attenuated tumor cell growth and proliferation, increased cell death, and enhanced oxidative stress, which together contributed to the antitumor response in vitro and in vivo [103]. It has been shown that the ACST2 blockade can impair T-cell homeostasis in Th1 and Th17 populations, but there was no effect of ASCT2 loss on initial T-cell activation, IL-2 secretion, Treg differentiation, or regulation of T or B cell populations. Since only the effects of chronic loss of ASCT2 were analyzed, it may not be possible to predict the immune effects induced by ASCT2 inhibitors [104].

### 3.3. Blockade of Immune Checkpoints by PDT Combined with Other Forms of Therapy

Knowledge of the immunosuppressive properties of the PD-1/PD-L1 axis is well established [105]. Not surprisingly, the desirable effects of PDT in anticancer therapy capable of affecting immune checkpoints and stimulating the immune response have been used to associate this method with other forms of therapy. Down-regulation of PD-L1 was achieved by Qiu et al. They designed a photodynamic immunostimulator by encapsulating a signal transducer and activator of transcription 3 (STAT3) inhibitor (Stattic) into a photosensitizer (protoporphyrin IX) and a modified PD-L1 blocking peptide (CVRARTR) by self-assembling the drug [106]. It is known that STAT3 is constitutively activated by abnormal tyrosine kinase activity in a broad spectrum of cancer cell lines and human tumors, and that its activity promotes PD-L1 expression and inhibits the immune response in breast cancer [107,108]. Previous studies have shown that the STAT3 inhibitor, Stattic, has the ability to induce apoptosis in cancer cells and that it enhances immunogenic cell death induced by chemotherapy [109]. The molecule developed by Qiu et al. can facilitate targeted drug accumulation in breast cancer cells with PD-L1 overexpression to block PD-L1 and inhibit STAT3 phosphorylation to down-regulate PD-L1. Moreover, through photodynamic therapy, it also increases intracellular oxidative stress, exhibiting potent antiproliferative activity, which also induces immunogenic cell death [107].

Jian et al. developed a new nanoparticle by combining the mitochondria-targeted heptamethrin-cyanine PDT dye MHI with tamoxifen via self-assembly with albumin. This strategy showed effective inhibition of PD-L1 protein expression and reversal of tumor hypoxia, which increased tumor T-cell infiltration and immune response, more effectively mitigating the development of lung metastasis [110]. This approach is particularly interesting, as it has previously been shown that tamoxifen administration for breast cancer treatment through direct action on myeloid-derived suppressor cells (MDSCs) enriched the population of leukocytes such as NK cells and NKT cells [111]. PD-1+ NK cells can be inhibited in killing cancer cells which means that PD-1 is an important checkpoint for NK cell activation, and PD-1 blockade can induce an antitumor response in these cells [112]. Moreover, MDSCs can activate the PD-1/PD-L1 axis, inducing a group of PD-1-PD-L1+ Bregs that exert immunosuppressive effects, so blocking MDSCs may provide additional benefits [58]. The association of this molecule with ICI could result in improved treatment efficacy, as tamoxifen has a role in TGF-β inhibition, and numerous preclinical and clinical studies have shown that overactive TGF-β signaling is closely associated with ICI resistance [110,113].

A completely new strategy was developed by Fang et al. by associating PDT with PS indocyanine green with a nanoplex to deliver small interfering RNAs (siRNA) oligo against siPD-L1 encapsulated MPM, in order to give it the ability to reach tumor tissues via Siglec-15, which is expressed on the membrane [114]. The ability of siRNA to silence specific genes has been demonstrated in mammals and anti-cancer therapies based on interfering RNA (RNAi) have been proposed in recent decades as a new technique for cancer treatment [115]. Currently, there are three approved siRNA drugs and seven others are in phase 3 clinical trials [116]. Fang et al. observed that synchronously delivered siPD-L1 attenuated the ROS-induced PDT-generated increase in PD-L1 immunosuppressive expression, thereby effectively inducing a potent antitumor immune response in a mouse model of breast cancer xenografts [114].

As mentioned earlier, there have been reports of PDT’s ability to raise PD-1 levels. Anand et al. show that the levels of cells expressing the PD-1 checkpoint receptor were reduced following vitamin D and PDT. Moreover, increased levels of damage-related molecular patterns and infiltration of neutrophils, macrophages, and dendritic cells were observed after combining PDT with vitamin D [37]. However, a recent study showed that vitamin D supplementation may have a bimodal function of increasing serum PD-L1 levels when serum PD-L1 levels are very low and decreasing serum PD-L1 levels when serum PD-L1 levels are very high. Presumably, vitamin D can increase the number of T cells by eliminating the suppressive effect of the PD-1 PD-L1 pathway on T cells [117]. Thus, in the context of this approach, it seems important to establish a limit at which PDT-reduced PD-L1 expression will not be increased by vitamin D.

He et al. developed a molecule containing MnO2, PS IR780, an indoleamine 2,3-dioxygenase inhibitor, and a paclitaxel dimer capable of reducing intra-neoplastic infiltration of M2-type TAMs, alleviating TME hypoxia, reducing PD-L1 expression of tumor cells, consequently eliminating primary tumors, and almost completely preventing tumor recurrence and metastasis of breast cancer [118]. The addition of anti-PD-1 to their strategy seems interesting, as the combination of PD-1 inhibitors and paclitaxel has previously been shown to be effective and may be a potential treatment as second-line therapy in advanced gallbladder cancer [119]. A new approach to the use of IDO inhibitors was proposed by Choi et al. They created multilayer structured upconversion nanoparticles for near-infrared light-induced PDT and immunotherapy combination therapy modified with folic acid, the photosensitizer chlorin e6, and 1-methyl-tryptophan as an indoleamine 2,3-dioxygenase inhibitor (Table 2). The presence of folic acid ensured targeting of cancer cells and the ROS produced led to their death and activation of immunogenic cell death [120].

**Table 2:** Summary of recent research papers describing PDT and immunological checkpoints used in this article.

Authors	Results
Gurung et al. [35]	PDT with PS Ce6 inhibit PD-1/PD-L1 axis and induce immune response.
Lobo et al. [36]	PDT with PS redaporphin stimulates CD80 overexpression on cancer cells.
Anand et al. [37]	PDT combined with vitamin D administration shows increased recruitment of immune cells and decreased levels of cells expressing the checkpoint receptor PD-1+.
Delcanale et al. [40]	PDT treatment efficacy does not correlate with PD-L1 expression levels on the surface of tumor cells.
Bhandari et al. [52]	PDT can simultaneously increase ⍺-PD-L1 antibody delivery and induce immunogenic cell death (ICD) in mouse head and neck AT-84 tumors.
Zheng et al. [54]	The new nanoparticle, Combo-NP, exhibits dual functionality by inducing ROS and ICD, leading to increased production of cytotoxic T lymphocytes (CTL) and normalization of tumor blood vessels, inhibiting tumor metastasis.
Sun et al. [62]	The combination of PDT, immune checkpoint blockers, and EV antigens can significantly improve the efficacy of cancer immunotherapy by improving immunosuppressive TME activation of tumor-specific T cells and reversing macrophage depletion.
Wang et al. [67]	Cascade-augmented nanoimmunomodulator with multienzyme-like activities, which includes superoxide dismutase, catalase, peroxidase, and glutathione oxidase, that dissociates under an acidic and abundant GSH TME, leads to stimulation of immune response in “cold” tumors, which may enhance ICI therapy.
Zheng et al. [70]	Novel nanoplatform using cascade of action of multiple enzymes in TME leads to stimulation of immune response in “cold” tumors, which may enhance ICI therapy.
Zhang et al. [73]	A novel therapeutic strategy based on PD-L1 aptamer-based spherical nucleic acids (SNAs) enables simultaneous PDT, chemotherapy, and enhanced immunotherapy, leading to effective inhibition of both primary and untreated distant colorectal tumors in mice, with minimal risk of immune-related side effects.
Feng et al. [74]	A new class of mitochondria-targeted and fluorinated polymers with aggregation-induced emission properties by controlling immune checkpoints in response to therapy may represent a promising strategy for cancer treatment.
Yao et al. [76]	An innovative therapeutic strategy based on a single-molecule prodrug combining acid-activated doxorubicin with aggregation-induced emission photosensitizer (AIEgen) combined with a caspase-3-responsive peptide enables the release of doxorubicin in acidic TME, activation of caspase-3 generation, and aggregation of photosensitizers in situ, leading to effective TNBC ICD and DC maturation, consequently supporting the efficacy of TNBC ICI immunotherapy.
Yang et al. [79]	The construction of albumin nanospheres containing photosensitizer-IR780 in the core and cGAS-STING/producer H2S-ZnS agonists on the shell (IR780-ZnS@HSA), through induction of pyroptosis, activation of the cGAS-STING signaling pathway, and stimulation of the immune response, can lead to inhibition of tumor growth and improved efficacy of anti-PD-L1 antibody immunotherapy, being an innovative therapeutic strategy in TNBC.
Qu et al. [87]	Nanoplatform based on midkine nanobodies and solid-state polymer nanoparticles can lead to inhibition of tumor progression and activation of antitumor immunity through local ROS generation, drug delivery, and activation of immune response and represents a promising strategy for precision photoimmunotherapy of pancreatic ductal adenocarcinoma.
Su et al. [90]	New Cu-MOF@RCD nanoreactors and anti-PD-L1 antibodies through a combination of PDT, PTT, CDT, glutathione depletion, and ICI eliminate primary tumors and inhibit the growth of untreated distant tumors and cancer metastasis.
Zhou et al. [93]	A photoresonant nanocomplex that self-assembles from BODIPY-modified poly(amidoamine) (BMP), enabling stable encapsulation of FSP1 inhibitor (iFSP1) and PS Ce6, can activate ferroptosis via PDT, inhibit ferroptosis suppressor protein 1 (FSP1), stimulate ICD, and thus enhance anti-PD-L1 ICI efficacy.
Zhu et al. [97]	Combination of PDT enhanced with di-iodinated IR780, liposome-containing ferroptase inhibitor liproxstatin-1, and modified photosensitizer Icy7 can induce ICD and inhibit tumor neutrophil ferroptosis, effectively inhibiting gastric cancer growth.
Gu et al. [98]	The functional nanoplatform, containing PS IR780 and 2,2′-azobis[2-(2-imidazolin-2-yl)propane]-dihydrochloride (AIPH), enables PTT and PDT generated by oxygen-independent ROS, which stimulates ICD and blocks immune checkpoints, shows potential in effectively targeting both primary tumors and distant metastases.
Zhao et al. [102]	A photodynamic immunostimulant called BVC, which reprograms tumor metabolism through inhibition of glutamine transport and GSH synthesis, leads to an increased immune response and blockade of the PD-1/PD-L1 immune checkpoint, which in turn leads to effective treatment of metastatic tumors.
Qiu et al. [106]	A photodynamic immunostimulator (PCS) designed to simultaneously down-regulate and block programmed cell death 1 (PD-L1) ligand in tumor cells enables effective inhibition of breast cancer growth by photodynamic therapy (PDT) and activation of the systemic immune response.
Jiang et al. [110]	Combining PDT with tamoxifen may be an effective approach to simultaneously reduce PD-L1 and TGF-β expression, which may improve the effectiveness of PDT.
Fang et al. [114]	Novel RNA interference (RNAi) therapy strategy by using biomimetic nanoplex with dual redox reactivity, enables efficient delivery of siRNA to cancer cells and improves photodynamic immunotherapy through PDT-induced self-synergistic immunogenic cell death.
He et al. [118]	A novel albumin nanoplatform containing MnO2 efficiently delivers PDT, including PS IR780, as well as NLG919 and paclitaxel dimer, providing a simultaneous solution to the problems of tumor hypoxia, enhancement of ICD induction, and modulation of immunosuppressive TME.
Choi et al. [120]	MSUCN nanocarriers that combine immunotherapy and photodynamic therapy, enabling synergistic anti-cancer therapy by actively targeting tumor cells with folic acid and stimulating the immune response by generating reactive oxygen species and inhibiting the IDO pathway.

### 3.4. Future Research Directions

Despite the impressive achievements of the research conducted to date, it is necessary to further expand the knowledge of PDT and immune checkpoints (Table 3). As mentioned in earlier sections, the basic issue of the effect of photodynamic therapy on the expression level of immune checkpoints has not been clarified precisely so far. The lack of this knowledge may significantly affect the effectiveness of the association of PDT with ICI. This is due to the fact that in anticancer therapy, on the one hand, it is desirable to reduce the number of PD-1/PD-L1 receptors, while on the other hand, it has been shown that the lack of reactive PD-L1 expression can mean a poor response to PD-1 blockade therapy. The association of PDT with CTLA-4 inhibitors also seems to be an interesting direction of research. So far, reports on this subject are very limited. As mentioned above, the efficacy of ICI delivery can be enhanced by affecting abnormal tumor vasculature. Thus, a solution to increase the efficacy of the PDT–ICI combination would be to develop a new nanoparticle or an optimal PDT regimen to destroy vessels without inducing thrombus formation. It is clear that an apt direction for research to improve the effects of cancer therapy is to increase the efficacy of PDT and to address such problems as the suboptimal properties of photosensitizers and the lack of available oxygen or its depletion, which prevents effective ROS generation. There is already work using associations of different drugs and strategies to improve photosensitizer delivery and break the hypoxic TME. When designing new molecules, attention should also be paid to immunosuppressive cells, which inhibit the immune response in multiple ways. A well-defined therapeutic regimen of PDT capable of most effectively inducing immunogenic cell death has also still not been established. In summary, future research directions should focus on elucidating the precise mechanisms by which PDT affects the PD-1/PD-L1 axis and on designing a molecule with optimal characteristics to improve the multidimensional efficacy of both PDT and ICI, including in combination with other forms of therapy, such as chemotherapy. Particularly promising seems to be the attempt to solve the above-mentioned problems using artificial intelligence, the application of which in medical research has grown rapidly in recent years, yielding promising results in the search for new molecules for therapy.

**Table 3:** Benefits and limitations of PDT depending on immunological checkpoints.

**Advantages**
Induction of immunogenic cell death and activation of antitumor immune response
Reduction in the negative impact of the immunosuppressive tumor environment on the effectiveness of the immune response
Reduction in the negative effect of immunosuppressive cells on the effectiveness of the immune response
Promotion of proliferation and intratumor infiltration of cytotoxic T cells by combining repetitive PDT with anti-PD-1 therapy
PDT’s ability to enhance delivery of anti-PD-L1 antibodies to the tumor
**Disadvantages**
Lack of a precisely defined PDT therapeutic regimen capable of most effectively inducing immunogenic cell death
No explanation of the exact mechanisms by which PDT affects the PD-1/PD-L1 axis
Lack of optimal PDT strategy to destroy vessels without simultaneous induction of thrombus formation

## 4. Conclusions

Based on recently published promising study results, it is suspected that the combination of photodynamic therapy and immune checkpoint blockers may solve the problem of treatment failure in at least some types of cancer. Importantly, this strategy offers the potential to cure metastatic lesions, and the addition of other molecules can provide thoughtful targeting of the tumor’s immunosuppressive environment and improve treatment efficacy. Achieving accelerated clinical translation of PDT and ICI combination therapy is possible by focusing on the future research directions listed above. It is important to determine the optimal doses and timing of their administration, and to prove the safety of such therapy, without which it is impossible to introduce PDT with ICI in the clinic. In order to introduce this combination in the clinic on a permanent basis, larger, multicenter clinical trials are needed to confirm the initial optimistic results and to accurately assess the efficacy and safety of combination therapies. It is clear that a deeper understanding of the mechanisms of action of PDT and ICI and their synergies has important implications for the clinical translation of the PDT–ICI combination. In conclusion, photodynamic therapy has the ability to affect the expression levels of immune checkpoints, and its association with their blockers and other molecules may be a promising direction in cancer treatment.

## Figures and Tables

**Table 1:** Inclusion and exclusion criteria.

**Inclusion**
Papers describing PDT and immunological checkpoints were included
Only original research papers were included
Only papers describing new strategies or reports in the field of PDT and immune checkpoints were included
Both in vitro and in vivo papers were included
**Exclusion**
Review papers were excluded
Articles in a language other than English or Polish were excluded
Papers written before 2023 were excluded

## Abbreviations

**Abbreviation**	**Definition**
AIPH	2,2′-azobis [2-(2-imidazolin-2-yl)propane]-dihydrochloride
cGAS/STING	Cyclic guanosine monophosphate/adenosine synthase/interferon gene stimulator
CTL	Cytotoxic T lymphocyte
DAMP	Damage-associated molecular patterns
DC	Dendritic cell
EV	Extracellular vesicles
FGFR	Fibroblast growth factor receptor
FSP1	Ferroptosis suppressor protein 1
ICD	Immunogenic cell death
IDO	Indoleamine 2,3-dioxygenase
ITIMs	Tyrosine inhibitory motifs
KIT	Type III receptor tyrosine kinase
MDSC	Myeloid-derived suppressor cell
PD-1	Programmed cell death protein 1
PDAC	Pancreatic ductal adenocarcinoma
PDGFRB	Platelet-derived growth factor receptor beta
PD-L1	Programmed death-ligand
PDT	Photodynamic therapy
PS	Photosensitizer
RET	Receptor-tyrosine kinase
ROS	Reactive oxygen species
siRNA	Short inhibitor RNA
STAT3	Signal transducer and activator of transcription 3
STING	Stimulator of interferon genes
TAMs	Tumor-associated macrophages
TDO	Tryptophan 2,3-dioxygenase
TGF-β1	Transforming growth factor β1
TME	Tumor microenvironment
VEGFR	Vascular endothelial growth factor receptor
